# Ingestion of Diazotrophs Makes Corals More Resistant to Heat Stress

**DOI:** 10.3390/biom12040537

**Published:** 2022-04-02

**Authors:** Valentine Meunier, Sophie Bonnet, Mercedes Camps, Mar Benavides, Jeff Dubosc, Riccardo Rodolfo-Metalpa, Fanny Houlbrèque

**Affiliations:** 1 ENTROPIE UMR 9220 (CNRS, IRD, UR, UNC, IFREMER) Institut de Recherche pour le Développement, Nouméa 98848, New Caledonia; riccardo.rodolfo-metalpa@ird.fr (R.R.-M.); fanny.houlbreque@ird.fr (F.H.); 2 Aix-Marseille University, CNRS, IRD, MIO UM 110, 13288 Marseille, France; sophie.bonnet@mio.osupytheas.fr (S.B.); mercedes.camps@mio.osupytheas.fr (M.C.); mar.benavides@mio.osupytheas.fr (M.B.); 3 Laboratory of Marine Biology and Ecology, Aquarium des Lagons, Noumea 98807, New Caledonia; jeff.dubosc@aquarium.nc

**Keywords:** coral, heat stress, coral bleaching, heterotrophy, diazotrophy, climate change

## Abstract

Over the past decade, coral bleaching events have continued to recur and intensify. During bleaching, corals expel millions of their symbionts, depriving the host from its main food source. One mechanism used by corals to resist bleaching consists in exploiting food sources other than autotrophy. Among the food sources available in the reefs, dinitrogen (N_2_)-fixing prokaryotes or planktonic diazotrophs (hereafter called ‘PD’) have the particularity to reduce atmospheric dinitrogen (N_2_) and release part of this nitrogen (diazotroph-derived nitrogen or DDN) in bioavailable form. Here, we submitted coral colonies of *Stylophora pistillata*, fed or not with planktonic diazotrophs, to a temperature stress of up to 31 ± 0.5 °C and measured their physiological responses (photosynthetic efficiency, symbiont density, and growth rates). Heat-unfed colonies died 8 days after the heat stress while heat-PD-fed corals remained alive after 10 days of heat stress. The supply of PD allowed corals to maintain minimal chlorophyll concentration and symbiont density, sustaining photosynthetic efficiency and stimulating coral growth of up to 48% compared to unfed ones. By providing an alternative source of bioavailable nitrogen and carbon, this specific planktonic diazotroph feeding may have a profound potential for coral bleaching recovery.

## 1. Introduction

The success of scleractinian corals in nutrient-poor waters surrounding coral reefs is due to their symbiosis with unicellular dinoflagellates belonging to the Symbiodiniaceae family [1]. These symbionts translocate a large fraction of photosynthetically fixed carbon (C) and amino acids to the host for its nutritional needs [2]. Coral reefs are experiencing increasingly frequent and devastating bleaching events [3]. During bleaching, corals expel millions of their symbionts and/or suffer a loss of photosynthetic pigments, depriving the host from its main nutrition source [4,5]. This process leads to coral death and to the decline of coral reefs if bleached corals are not able to rapidly recover their symbionts [3]. Bleaching susceptibility varies among species, depths, and locations (e.g., [6,7,8,9]), and is also influenced by coral morphology [10,11], physiological responses of both animal and symbionts [12,13], and symbiont types hosted by corals (e.g., [14,15,16]).

Another mechanism used to resist and recover from bleaching consists in exploiting food sources other than autotrophy. Indeed, corals are voracious predators that can feed on a wide range of prey ranging from pico-nanoplanktonic cells (with a size < 20 µm; [17]) to macrozooplankton [18,19]. Multiple laboratory experiments have shown that heterotrophy increases skeletal and tissue growth [17,20,21,22,23], allows corals to build up energy reserves [24,25], increases fertility [26], and reduces sensitivity to acidification [27,28,29]. Coral ability to switch from an autotrophic to a heterotrophic diet by increasing their feeding rates on zooplankton [22,30,31], making them more resistant to bleaching [30,32,33,34], has been much less studied. Only two studies have shown that corals increase their consumption of pico-nanoplankton during heat stress [35,36]. Among these small preys, dinitrogen (N_2_)-fixing prokaryotes (subsequently referred to as planktonic diazotrophs, hereafter called ‘PD’) have received little attention. Many coral reef ecosystems are characterized by high planktonic diazotroph abundance and activity; they are very widespread in the Western South Pacific (e.g., New Caledonia, Papua New Guinea, and the Australian Great Barrier Reef) [37,38,39,40] but also in Hawaii, in the Caribbean, and the Red Sea [41,42,43]. In New Caledonia, planktonic diazotrophs support ~80% of the primary production of other phytoplankton in summer [44,45]. The lagoon is dominated by picoplankton (non-diazotrophic, i.e., *Synechococcus* and *Prochlorococcus*) but the diazotrophs, even if they are less abundant, are much larger (nanoplankton and microplankton size) and thus represent an important part of the total biomass [46]. Planktonic diazotrophs reduce atmospheric N_2_ into bioavailable ammonium (NH_4_^+^), and release part of the recently fixed nitrogen (Diazotroph-Derived Nitrogen, DDN) in seawater, providing sufficient nitrogen stocks for the development of the planktonic food web in oligotrophic waters [47]. These planktonic diazotroph cells are thus, by definition, very rich in nitrogen [48,49]. Their ingestion by scleractinian corals was first demonstrated by [50] using the ^15^N_2_ labelling method. Direct consumption of planktonic diazotrophs would provide 0.76 ± 0.15 μg N cm^−2^ h^−1^ to corals, which corresponds to six times the daily N supply by non-diazotrophic pico- and nanoplankton [17]. Bleached colonies of *S. pistillata* incorporate more PD than healthy colonies [36], but the consequences on coral metabolism are not known. By providing an alternative source of bioavailable N and C, this increased incorporation of PD may have a profound influence on coral bleaching recovery.

To improve our understanding of the physiological response of corals to heat stress in anticipation of more frequent and severe coral bleaching events, we investigated if and how nutrition on PD is involved in the resistance to high temperature of the coral species *S. pistillata*. We evaluated key physiological measurements (photosynthetic efficiency, growth rates) and tissue parameters (chlorophyll, protein concentrations, and symbiont densities) of colonies of *S. pistillata*, fed or not with PD during heat stress.

## 2. Materials and Methods

### 2.1. Diazotrophic Strains Acclimation

Before the experiment, 4 strains of PD isolated from the Western Tropical South Pacific (WTSP) (details in Table 1) were grown in an F/2 culture medium [51] modified without nitrate to ensure obligate diazotrophy. This N-free F/2 was made with 0.2 µm-filtered sterilized seawater collected in the New Caledonian Lagoon (the provenance of studied corals, 166.44° E, 22.48° S), to prevent any physiological stress when added to aquaria containing corals colonies. Strains were cultivated in 2 L Erlenmeyer flasks in semi-continuous conditions, i.e., they were maintained under an exponential growing phase for the entire duration of the experiment (21 days), by adding 300 mL of new medium every 2 days. In vivo chlorophyll, fluorescence was measured every 3 days (model Trilogy, Turner Designs). A mixture of these 4 strains was given to some of the corals (see description of the protocol below).

### 2.2. Coral Preparation and Acclimation

One hundred and sixty terminal branch portions (3–5 cm) of *S. pistillata* were cut from 20 different parent colonies in the New Caledonian lagoon (22°21′339″ S; 166°15′404″ E; sampling license issued by the ‘Province Sud’, Government of New Caledonia). Coral fragments were transported from the collection site in a cooler and randomly assigned experimental tanks. They were hung on nylon wires and suspended in eight 6.5 L aquaria (around 20 coral fragments per aquarium), spaced apart from each other without ever touching. All physicochemical parameters (temperature, salinity, nutrient concentration, and pH) were rigorously controlled in each tank so that the only difference between the tanks was temperature. Therefore, despite the fact that this setup could be considered as a pseudoreplication, the results obtained are representative, and the most parsimonious explanation for the differences observed between the coral batches is clearly the feeding and not stochastic errors. Fragments were maintained in an open seawater system, continuously supplied with 40 µm-filtered seawater, renewed at a rate of 16.5 L h^−1^, and mixed using a submersible pump (Aquarium system, micro-jet MC 320, Mentor, OH, USA). In each tank, coral colonies were spaced apart and never touched each other. Temperature (28 ± 0.2 °C) and salinity (35.80 ± 0.01) were kept constant using heaters connected to electronic controllers and monitored with a YSI MPS 556 probe (YSI, Yellow Springs, OH, USA). Corals received a constant irradiance of 120 ± 10 μmol photons m^−2^ s^−1^ (photoperiod 12 h:12 h light:dark) using four Aquablue plus neon bulbs (blue–white, 15,000 K, Giesemann, Germany). All coral fragments were left to acclimate and grow in these conditions for three weeks before starting coral feeding and carrying out the first sampling of the colonies (T0 sampling).

### 2.3. Experimental Setup and Conditions

**Figure 1:**
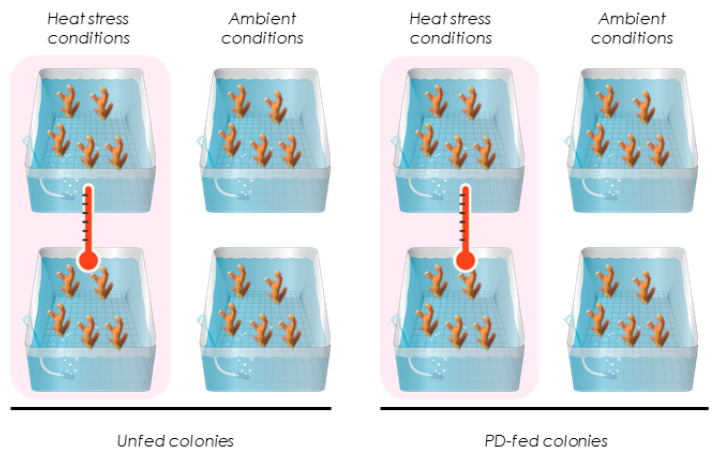
Experimental design with two feeding regimes. Each condition was duplicated with 20 fragments per tank. Ambient conditions of 28 ± 0.2 °C. Heat stress conditions with a temperature increase of up to 31 ± 0.5 °C.

After T_0_ and during the 55 days of the experiment, the environmental parameters (temperature, irradiance) were the same as those described above and were measured in each of the 8 aquaria. In 4 aquaria, coral fragments were not fed (called unfed corals), while in the other 4, coral fragments were fed exclusively with PD (called PD-fed corals) (Figure 1). These corals were fed 3 times/week with a mix of the 4 strains of PD. To prepare the PD ‘solution’, the mix of the 4 strains (100 mL) was first centrifuged at 5000 × *g* for 4 min 30 s to obtain a pellet and remove the entire culture medium. The pellet was then gently resuspended in 0.2 μm-filtered seawater. Between 100 and 200 mL of strain mix were added in each tank to reach final concentrations of 10^3^ and 10^6^ per mL, corresponding to the average concentration measured in the NC lagoon [37,52,53]. Each feeding episode was carried out for 2 h after the lights were switched off. Seawater circulation in the tanks was cut off during this time to avoid the flushing of prey. Both PD-fed and unfed corals were maintained in these conditions for 55 days before they were exposed for 10 additional days to a gradual increase in temperature of 0.3 °C per day until reaching 31 ± 0.5 °C on day 65 of the experiment (hereafter called T_65_). The same feeding protocol was used throughout the experiment, before and during the heat stress. PD-fed and unfed corals submitted to heat stress are referred to as ‘heat-PD-fed’ and ‘heat-unfed’ corals, respectively. PD-fed and unfed corals maintained at the initial temperature of 28 ± 0.2 °C are referred to as ‘PD-fed’ and ‘unfed’ corals under ambient conditions. For each measured parameter, 6 coral fragments per time (T_0_ and T_65_) and per condition were sampled. Therefore, measurements of photosynthesis, Symbiodiniaceae densities, chlorophyll, and protein concentrations were performed on the same coral fragment.

### 2.4. Symbiodiniaceae Density and Total Chlorophyll, and Protein Content

Six coral fragments per time (T_0_ and T_65_) and per condition were sampled. Tissue was removed from the skeleton using an air pick and homogenized with a Potter tissue grinder [54,55]. Symbiont cells were counted 3 times with light microscopy using a Neubauer’s cell. Subsamples of 10 mL from each coral tissue solution were centrifuged at 8000× *g* for 10 min at 4 °C, and the supernatant was removed. The pellet containing the symbiont was resuspended in 100% acetone for 24 h (4 °C) in the dark to extract chlorophyll *a* and *c*_2_. The extracts were centrifuged at 10,000× *g* for 15 min and absorbances were read at 630, 663, and 750 nm. Chlorophyll *a* and *c*_2_ concentrations were computed according to the spectrometric equations from [56,57]. Chlorophyll *a* and *c*_2_ are given as total chlorophyll. All measurements were normalized to the skeletal surface area (cm^2^), estimated using the paraffin wax-dipping method [58,59]. Sub-samples were taken at the beginning and after 30 days of experiments to assess the total protein content (mg prot cm^−2^) according to [60] and [57] with a BCAssay Kit (Uptima, Interchim).

### 2.5. Growth Rates

Growth rates were monitored every 15 days on every coral fragment using the buoyant weight (BW) technique [61]. BW gives an estimation of the calcification rates of corals over long time scales and considers both daytime and nighttime calcification rates. Samples hung on a nylon wire were weighed using a Mettler XP205 electronic balance (readability of 0.01 mg) in seawater of known density, and temperature and salinity were continuously measured. The net BW of the corals was converted into dry weight using the density of pure aragonite (2.94 g cm^−3^). Their growth rates were calculated as the change in dry weight between the initial and the final weight and expressed in mg g^−1^ d^−1^.

### 2.6. Photosynthetic Efficiency Measurements

Photosynthetic efficiency (Fv/Fm) and the electron transport rate (ETR) of the Photosystem II (PSII) of symbiont in hospite were measured at T_0_ and at T_65_ using a DIVING-PAM fluorometer (DIVING-PAM, Walz, Germany [62]). Measurements were performed on 12 coral fragments for each condition. Before the measurements, these coral fragments were dark-adapted for 15 min (after [63]). The 8 mm optical fiber was maintained perpendicular to the fragments’ surface using a black-jacket at a fixed distance of 5 mm guaranteeing a correct distance of the optical fiber from the coral. A weak-pulse red light (max. intensity < 1 mol photon m^−2^ s^−1^, width 3 µs, frequency 0.6 kHz) and a saturating pulse of actinic light (max. intensity 8000 µmol photon m^−2^ s^−1^, pulse width 800 ms) were applied to the coral surface to measure the minimal (F_0_) and maximal (F_m_) Chl a fluorescence, respectively. ETR was obtained through the rapid light curves (RLCs). RLCs were collected by illuminating corals for 10-s periods with eight actinic light sequences from 0 to ca. 1500 µmol photons m^−2^ s^−1^. Variable fluorescence (F_v_) was calculated resulting in Fm-F0 [64] and F_v_/F_m_ values that may be used as a quantitative measure of photo-inactivation during coral bleaching [16]. F_v_/F_m_ values of healthy corals ranged between 0.5 and 0.75 [65]. After the physiological measurements, corals were rinsed 6 times with filtered seawater to remove cells that potentially adhered to the coral surface [20], transferred to zip-lock bags, and stored at –20 °C until analysis.

### 2.7. Statistical Analyses

All tests were performed using R version 4.1.2 within RStudio (Version 2021.09.1+372). The non-parametric Mann–Whitney–Wilcoxon test was first used to test for significant differences in Symbiodiniaceae densities and total chlorophyll content between feeding regimes. A three-way ANOVA was performed to assess the differences in the measured parameters (photosynthetic efficiency, growth rates, Symbiodiniaceae densities, chlorophyll, and protein concentrations). The tank effect was included as a random factor nested in the feeding regime, with temperature treatment and feeding regime as a fixed factor. The tank effect was eliminated from the ANOVA analyses because it was not significant for all measured parameters. Therefore, data between the tanks were pooled and two-way ANOVAs were used to test the effects of the feeding regime and temperature on symbiont density, chlorophyll concentration, growth rate, photosynthetic efficiency (F_v_/F_m_), and electron transport rate (ETR). When the ANOVA determined a significant difference, a Tukey’s honest significant difference test (HSD) was used to test for pairwise differences while taking the interactions between the different variables into account. The *ggplot2* package [66] was used to create the box plot figures. Throughout the manuscript, values given are expressed as mean ± SE. Statistical significance was accepted at *p* < 0.05.

## 3. Results

### 3.1. Symbiodiniaceae Density, Chlorophyll, and Protein Concentrations

**Figure 2:**
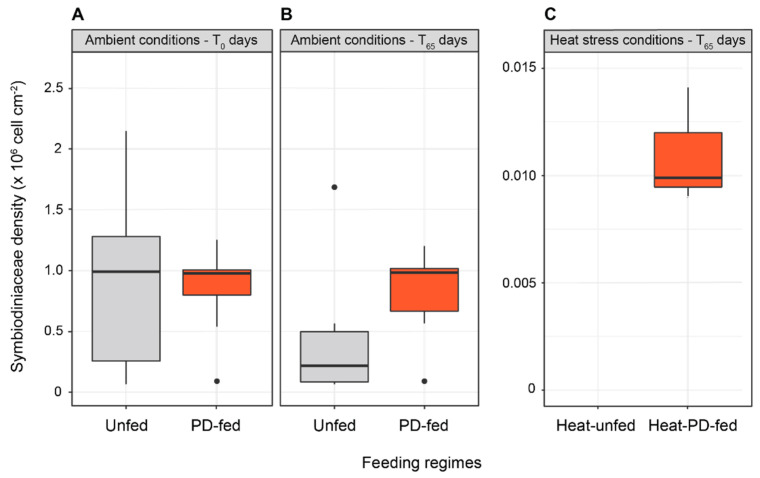
Symbiodiniaceae density (cell cm^−2^) in unfed and PD-fed colonies of *Stylophora pistillata* at the beginning of the experiment (T_0_) (**A**) and at 65 days of the experiment under (**B**) ambient and (**C**) heat stress conditions (N = 6; mean ± SE for each condition).

**Figure 3:**
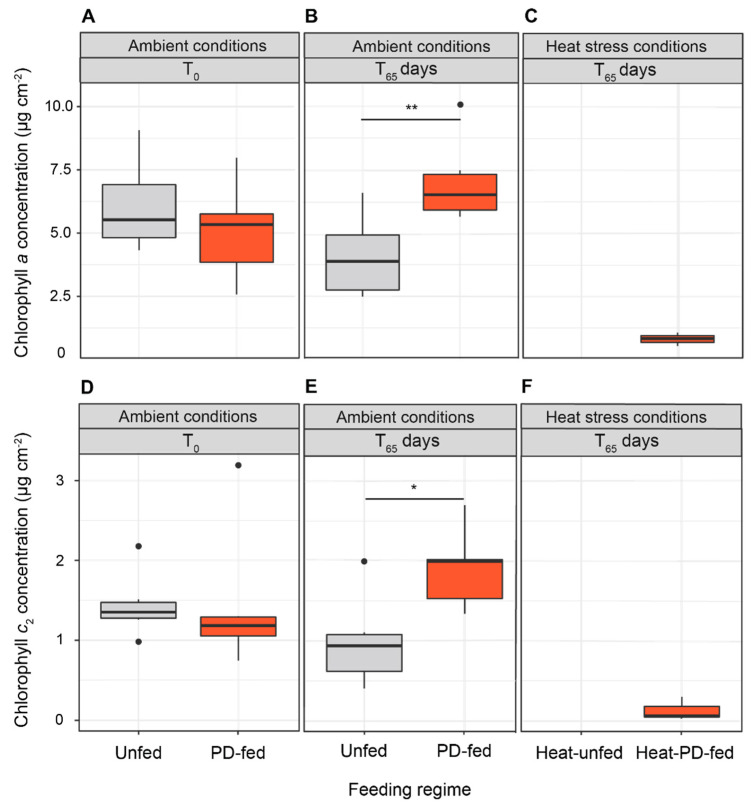
Chlorophyll *a* (**A**–**C**) and *c*_2_ (**D**–**F**) concentrations (µg cm^−2^) in unfed and PD-fed colonies of *Stylophora pistillata* at the beginning of the experiment (T_0_) and at 65 days of the experiment under ambient and heat stress conditions (mean ± SE; N = 6). Asterisks indicated significant differences between treatments (* *p* < 0.05; ** *p* < 0.01).

All the coral fragments remained healthy during the 55 days prior to heat stress. At T_0_ and T_65_ of incubation under ambient conditions, coral fragments had similar symbiont densities (8.78 ± 1.06 × 10^5^ cell cm^−2^; N = 6 for each tank) regardless of the feeding regime (Figure 2A,B). At T_0_, total chlorophyll concentrations (*a* + *c*_2_) were similar (7.109 ± 0.705 µg cm^−2^; N = 6 for each tank) for unfed and PD-fed corals (7.109 ± 0.705 µg cm^−2^; N = 6 for each tank) (Figure 3A,D); however, at T_65_ in ambient conditions, these concentrations decreased by 33% in unfed corals and conversely increased by 37% in PD-fed corals (two-way ANOVA, Tukey’s HSD test, ajd. *p*-value = 0.01) (Figure 3B,E).

Heat-unfed colonies died 8 days after the heat stress, which did not allow us to measure the related symbiont densities and chlorophyll concentrations. In contrast, heat-PD-fed corals remained alive after 10 days of heat stress with their symbiont densities and total chlorophyll concentrations decreased by 88% (0.925 × 10^6^ cell cm^−2^) and by 90% (0.973 µg cm^−2^), respectively (Figure 2C and Figure 3C,F).

At T_0_ and after 30 days of incubation under ambient conditions, coral fragments showed similar protein concentrations (0.802 ± 0.139 mg prot cm^−2^ and 0.751 ± 0.171 mg prot cm^−2^, respectively; N = 12) regardless of the feeding regime (two-way ANOVA, Tukey’s HSD test, ajd. *p*-value = 1; N = 6 for each feeding regime). As specified above, heat-unfed coral colonies died 8 days after the heat stress, leaving us no time to measure protein concentrations.

### 3.2. Growth Rates

**Figure 4:**
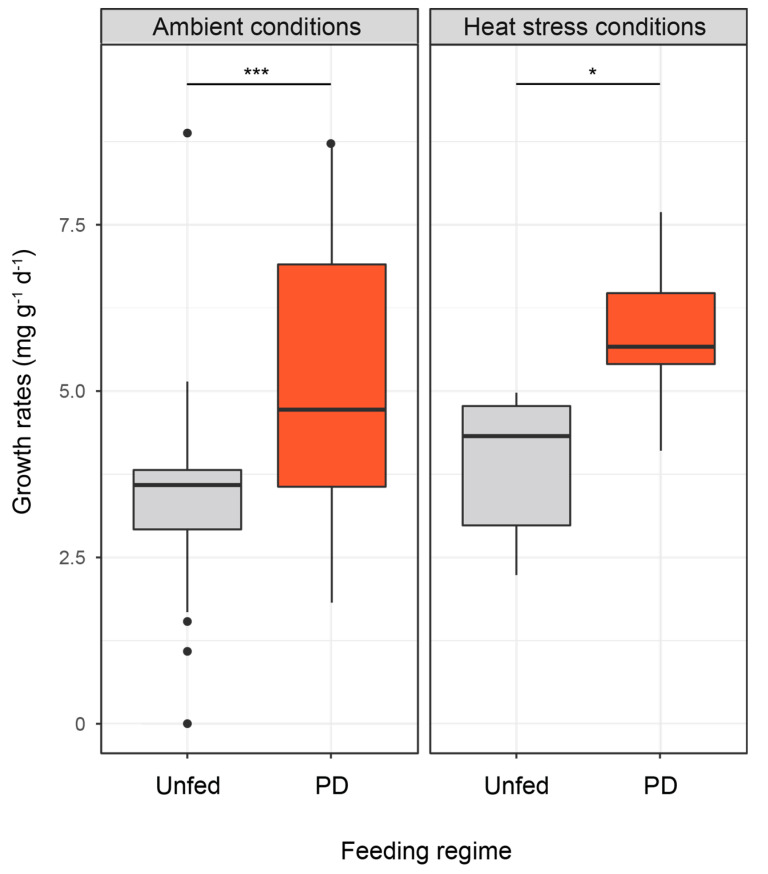
Growth rates of unfed (N = 40) and PD-fed (N = 44) colonies of *Stylophora pistillata* exposed for 55 days to ambient conditions and 10 days to heat stress conditions (mean ± SE). Asterisks indicated significant differences between treatments (* *p* < 0.05; *** *p* < 0.001).

At T_30_ under ambient conditions and 25 days prior to heat stress, PD-fed corals showed higher growth rates than unfed corals (5.2732 ± 0.2787 mg g^−1^ d^−1^ and 3.4362 ± 0.2745 mg g^−1^ d^−1^, respectively, two-way ANOVA, Tukey’s HSD test, ajd. *p*-value = 0.02). At T_65_ under ambient conditions, PD-fed coral growth rates were 48% higher than unfed corals (two-way ANOVA, Tukey’s HSD test, ajd. *p*-value = 0.0002). At T_65_, heat-PD-fed and PD-fed growth rates remained similar. Heat-PD-fed coral growth rates were still higher than those of heat-unfed corals, before the death of the latter 8 days after heat stress (two-way ANOVA, Tukey’s HSD test; ajd. *p*-value = 0.03) (Figure 4).

### 3.3. Fv/Fm and Electron Transport Rates of Symbiodiniaceae

At the beginning of the experiment, corals had similar F_v_/F_m_ (0.696 ± 0.016). At T_65_ in ambient conditions, the F_v_/F_m_ values remained unchanged regardless of the feeding regime (0.697 ± 0.014 for unfed corals; 0.689 ± 0.024 for PD-fed corals). However, Fv/Fm values of all coral colonies were impacted by the heat stress and significantly decreased by 29.5% and 28.3% for unfed and PD-fed corals, respectively.

**Figure 5:**
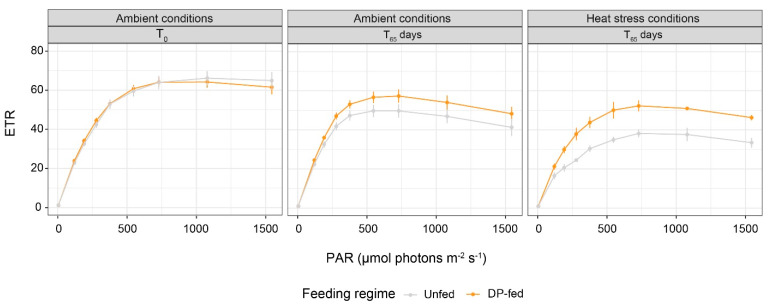
Electron transport rate (ETR) versus irradiance (PAR) for unfed and DP-fed colonies of *Stylophora pistillata* at the beginning of the experiment (T_0_) and at 65 days of the experiment under ambient and heat stress conditions (mean ± SE; N = 12).

At T_65_ in ambient conditions, ETR_max_ values were 1.15-fold higher in PD-fed corals compared to those of the unfed ones. After the heat stress, a decrease in ETR curves was observed in all coral colonies regardless of the feeding regime, but ETR_max_ values decreased more for heat-unfed corals (23%) than for the heat-PD-fed corals (9%) (Figure 5).

## 4. Discussion

Some coral species are able to increase their heterotrophic nutrition during bleaching episodes, which supplies their energy reserves, providing them with greater resistance, improved chances of survival [25,30,34,67,68,69], and allows them to recover their symbionts [70,71]. These conclusions were drawn by focusing primarily on the ingestion of mesozooplankton, and no study had so far investigated smaller planktonic fractions. Our experiment provides the first evidence that heat-stressed PD-fed corals are able to retain part of their symbionts and chlorophyll and appear more resistant compared to heat-stressed unfed ones, which died after only 8 days. We also show that a supply in planktonic diazotrophs (particularly rich in N) also alleviates some of the negative effects of heat stress on coral growth and photosynthetic efficiency.

In ambient conditions, feeding on planktonic diazotrophs induced significant changes in most coral physiological parameters. After 9 weeks of a planktonic diazotrophs’ diet, colonies significantly increased their chlorophyll contents. Corals benefit from these planktonic diazotrophs, directly either by grazing them [36,50], and/or indirectly by uptaking the DIN (dissolved inorganic nitrogen: NH_4_^+^ and DON) they released in surrounding waters [49,72,73]. This stimulation of the symbiotic compartment has been highlighted before for mesozooplankton-fed corals (e.g., [20,74,75,76]). As heterotrophy results in a significant release of NH_4_^+^ and as this NH_4_^+^ accumulates 14 to 23 times faster in the Symbiodiniaceae than in animal cells [77,78,79], planktonic diazotrophs’ diet stimulated pigment synthesis and symbiont division. The richness of planktonic diazotroph cells in vitamin B12 [80], which is an essential nutrient for the coral holobiont [81], has also contributed to this stimulation of the symbiotic compartment. In our study, ETRmax and Fv/Fm values of fed corals were similar to those of the unfed ones, and not higher as it has been demonstrated in mesozooplankton-fed corals (e.g., [22,76]). The PD-fed corals having higher symbiont densities should result in higher photosynthetic rates per surface regardless [21,22,71,82]. Given the N richness of diazotrophic cells, this specific diet should modify the photosynthate quality, with the highest amounts of N-rich amino acids [83].

One of the major results of this study is that an exclusive nutritional diet of planktonic diazotrophs stimulated coral growth by 48% compared to the unfed corals. Our data confirm that feeding enhances skeletal growth, suggesting that corals allocate a high proportion of the energy brought by planktonic diazotrophs to skeleton growth processes [75,84,85,86]. Skeleton growth is a dual process, involving the secretion of an organic matrix and the deposition of a CaCO_3_ fraction. The contribution of diazotrophic plankton, particularly rich in N, could thus participate in a stimulation of the production of this organic matrix, mainly composed of proteins, glycoproteins, and polysaccharides [87,88,89], as already demonstrated in mesozooplankton-fed corals [75]. This growth stimulation by planktonic diazotrophs could also be achieved indirectly by boosting photosynthesis as stated above. These two stimulating ways make that growth rates in fed corals, submitted to heat stress, remain stable and always higher than those of unfed ones in the same conditions. In [71], calcification rates of *S. pistillata* colonies fed for 9 weeks with *Artemia salina* were reduced by up to 65% compared to control temperatures. Unlike *A. salina* nauplii, planktonic diazotrophs release large amounts of NH_4_^+^ and DON into the surrounding water [49,72,73]. This surplus of N provided by the planktonic diazotrophs might have reduced calcification sensitivity to heat stress, as demonstrated for corals submitted to moderate nutrient inputs [90,91].

In corals under heat stress, the supply in planktonic diazotrophs helped maintain a minimal chlorophyll concentration and symbiont density to prevent a large decrease in the RLC curves, and above all, the colonies’ death. A decrease in chlorophyll content and symbiont densities was observed in heat-PD-fed corals (decrease of respectively 85% and 88%) but contrary to heat-unfed corals, all heat-PD-fed corals stayed alive, managed to keep some of their symbionts and pigments, and grew faster. As in the studies testing the effects of the ingestion of *Artemia salina* nauplii on the same species (*S. pistillata*; [21,22,92]), planktonic diazotrophs helped maintain photosynthetic efficiency of PSII. The input of this new N might have (*i*) facilitated the protein repair and re-synthesis of the PSII D1 protein (reviewed by [92,93]), (*ii*) increased the photosynthetic and photoprotective pigment (such as xanthophyll and peridinin) contents in coral tissue as in corals submitted to a moderate DIN enrichment [91], and/or (*iii*) reduced the degradation of the symbionts by increasing the synthesis of antioxidant compounds or heat-shock proteins [92,94].

Our results clearly show a positive effect of planktonic diazotroph feeding under heat stress, sustaining photosynthetic efficiency and growth of *S. pistillata* colonies. So far, heterotrophic feeding on planktonic diazotrophs has been little considered compared to the N supply provided by coral-associated diazotrophs. However, in the case of heat stress, Ref. [95] have recently shown that this N supply by coral-associated diazotrophs is compromised. A 10-day heat stress caused a change in coral-associated diazotroph communities, which led to a 30% decrease in fixed N. Conversely, our previous works highlighted that N supply by planktonic diazotrophs (*i*) can fulfill a large part of the coral N requirements, compared to the symbiotic fixation ([50,69,96,97]) and (*ii*) is significant for several coral species [69]. This last study finally demonstrates that, contrary to endosymbiotic diazotrophs, a supply of planktonic diazotrophs allows corals to be more resistant to bleaching. It is therefore particularly essential to consider the contribution of DDN by planktonic diazotrophs in the coral holobiont N cycling which appears to be one of the main strategies for coral recovery facing bleaching. In the context of climate change, marine heat waves are becoming more intense and frequent and coral reefs are among the most vulnerable ecosystems to this emerging threat [98,99]. Our very encouraging results, obtained in this study, imply that coral reefs where planktonic diazotrophs are abundant (e.g., Papua New Guinea, New Caledonia) could be more resistant to climate change. A supply of planktonic diazotrophs on the reefs could be included among innovative management approaches to improve the resilience of coral reefs with high conservation potential, during and after bleaching episodes [100,101].

## Figures and Tables

**Table 1:** Details of the 4 cultures of planktonic diazotrophs isolated from the Western Tropical South Pacific.

Genus	Morphological Type	Size	Location
*Xenococcus* spp.	Unicellular	∼6 µm	Noumea lagoon
*Cyanothece* spp.	Unicellular	∼5.5 µm	Noumea lagoon
*Calothrix* spp.	Filamentous heterocystous	∼50 µm	WTSP Ocean
*Rivularia* spp.	Filamentous heterocystous	∼30 µm	WTSP Ocean

## Data Availability

The original contributions presented in the study are included in the article, further inquiries can be directed to the corresponding author/s.
